# SOCS1 blocks G1-S transition in hepatocellular carcinoma by reducing the stability of the CyclinD1/CDK4 complex in the nucleus

**DOI:** 10.18632/aging.102865

**Published:** 2020-02-25

**Authors:** Jun Ding, Kangdi Xu, Suwan Sun, Chao Qian, Shengyong Yin, Haiyang Xie, Lin Zhou, Shusen Zheng, Wei Zhang

**Affiliations:** 1 Division of Hepatobiliary and Pancreatic Surgery, Department of Surgery, First Affiliated Hospital, School of Medicine, Zhejiang University, Hangzhou, Zhejiang Province, China; 2 Key Laboratory of Combined Multi-Organ Transplantation, Ministry of Public Health, Hangzhou, Zhejiang Province, China; 3 Key Laboratory of the Diagnosis and Treatment of Organ Transplantation, CAMS, Hangzhou, Zhejiang Province, China; 4 Key Laboratory of Organ Transplantation, Hangzhou, Zhejiang Province, China; 5 Collaborative Innovation Center for Diagnosis Treatment of Infectious Diseases, Hangzhou, Zhejiang Province, China; 6 State Key Laboratory for Diagnosis and Treatment of Infectious Diseases, First Affiliated Hospital, School of Medicine, Zhejiang University, Hangzhou, Zhejiang Province, China; 7 National Clinical Research Center for Infectious Diseases, Hangzhou, Zhejiang Province, China; 8 Ningbo Medical Center LIHUILI Hospital, Ningbo, Zhejiang Province, China

**Keywords:** hepatocellular carcinoma (HCC), suppressor of cytokine signalling 1 (SOCS1), cell proliferation, cell cycle, CyclinD1

## Abstract

Inhibitors of the CDK family of proteins have been approved for the treatment of a variety of tumours; however, the development of new drugs administered in combination with CDK inhibitors is expected to improve the therapeutic effect. We identified the function of suppressor of cytokine signalling 1 (SOCS1) in hepatocellular carcinoma (HCC) cell models and the xenograft mouse model. When SOCS1 expression was artificially upregulated, HCC cell lines were arrested at the G1-S transition in the cell cycle. Interestingly, during this process, total CyclinD1 protein increased, but the effective proportion decreased. We found that the deficiency of CyclinD1 in the nucleus is probably due to the decrease in the stability of nuclear CyclinD1 caused by the ubiquitin-based degradation of P21, thus inhibiting the progression of the cell cycle to S phase. After P21 expression was increased, the levels of the component that inactivates CyclinD1 decreased as expected. It showed that P21 has a partial promoting effect on cancer. SOCS1 is a good indicator of prognosis, tumour size and long-term survival after resection. SOCS1 is expected to become a drug target in combined with CDK family inhibitors.

## INTRODUCTION

In 2018, liver cancer became the sixth most common cancer in the world and the fourth highest cause of cancer-related death, with approximately 841000 new cases and 782000 deaths each year. Hepatocellular carcinoma (HCC) accounts for 75 to 85 percent of primary liver cancers diagnosed [1]. To date, surgical resection of small isolated HCC has achieved a five-year survival rate of more than 50% [2]. However, most patients with HCC have middle or advanced stage disease at the time of diagnosis. Some of these patients are eligible for liver transplantation, and the health care centres that perform these procedures have safely and effectively expanded liver transplantation candidates on the basis of Milan standards [3]. Unfortunately, there are still many patients with inoperable cancer, but the advent of molecular targeting therapies in recent years has greatly improved the overall survival of these patients [4]. Therefore, it is of great significance to develop more effective molecular targeting drugs in combination with current treatment methods to improve the curative effect and prolong the survival of HCC patients.

Cell cycle dysfunction is a common feature of human cancers [5]. Among the proteins involved in cell cycle progression, CyclinD1 mainly activates and binds the cyclin-dependent kinase CDK4, which is unique to G1 phase (pre-DNA synthesis). This kinase then phosphorylates a G1 inhibitor protein (Rb), which dissociates from the E2F transcription factor; E2F then promotes cell cycle progression from G1 phase to S phase [6]. CDK inhibitors play an important role in cell cycle control and are also a promising field of targeted cancer treatments [7]. Although several combination therapy strategies that have been studied, whether CDK4/6 inhibitors are more effective in combination with other treatments than as a monotherapy is an urgent problem yet to be solved.

SOCS1 is a member of the SOCS family of proteins, which are involved in the regulation of the JAK/STAT pathway by the classical negative feedback system. Similar to all SOCS family proteins, SOCS1 has a conserved carboxy terminal domain called the SOCS box and a central SH2 domain [8]. The SH2 domain of SOCS1 and the additional 12 amino-acid N-terminus adjacent to the domain are necessary for SOCS1 binding to the activated Jak kinase. In addition, the 12 amino-acid N-terminus is necessary to effectively inhibit the activity of Jak kinase [9, 10]. Biochemical binding studies show that the SOCS box of SOCS1 interacts with a component of the ubiquitin proteasome pathway and activates an E3 ligase [11–13].

In this paper, we determined that SOCS1 blocks the cell cycle progression of HCC cells in vitro and then explored the mechanism by which SOCS1 exerts its function through various in vivo and in vitro experiments. It was incidentally found that P21, well-known anticancer factor, also partially promoted cancer progression. Finally, we discuss the overarching effect of cancer in the current literature as it pertains to our work, as a single gene can determine not only the life and death of cancer but also the interdependence and interaction of different signalling systems. These cascading interactions are why we are looking for multi-disciplinary or combination treatments to combat cancer.

## RESULTS

### SOCS1 expression was decreased in HCC

**Figure 1:**
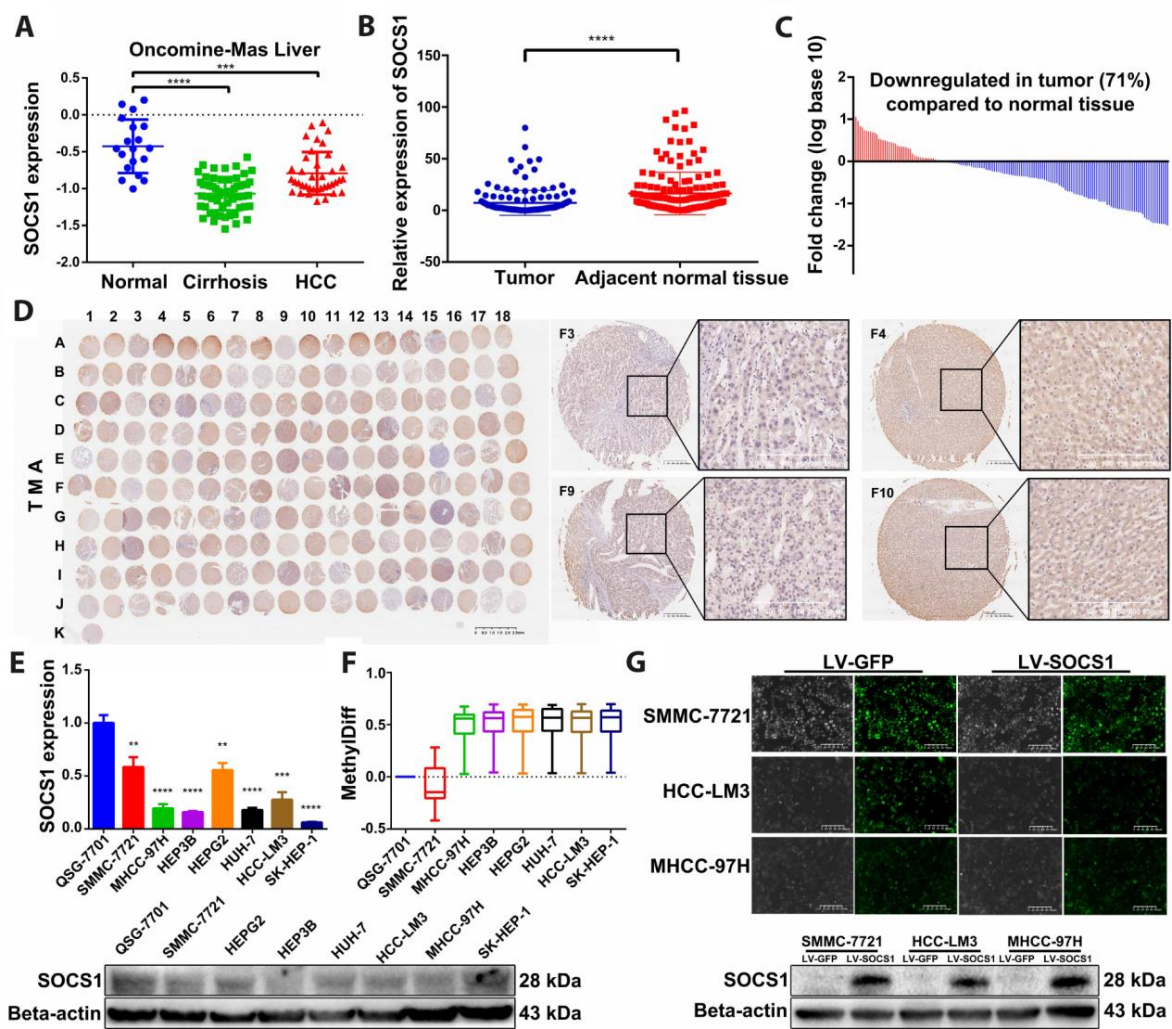
**SOCS1 expression levels in HCC and normal tissues.** (**A**) Oncomine-Online Platform (https://www.oncomine.org) showed reduced SOCS1 expression in HCC and paired normal liver tissues. ***P <0.001, ****P <0.0001. (**B**, **C**) In 159 pairs of tissues, SOCS1 expression was apparently downregulated in tumours compared with adjacent liver tissues. ****P <0.0001. In the majority of HCC tissues (71%), SOCS1 mRNA levels were reduced. (**D**) Immunohistochemical analysis of SOCS1 expression in 90 paired HCC (Odd number) and normal liver tissues (Even number). (**E**) The level of SOCS1 mRNA and protein expression was reduced in SMMC-7721, MHCC-97H, Hep3B, HepG2, Huh-7, HCCLM3 and SK-Hep-1 cells. ** P <0.005, ***P <0.001, ****P <0.0001. (**F**) The level of SOCS1 methylation increased in most HCC cell lines. (**G**) SMMC-7721, HCC-LM3 and MHCC-97H cells showed successful stable overexpression of SOCS1.

To observe the difference in SOCS1 expression between normal liver tissue and HCC, we verified SOCS1 expression in the Oncomine-Online Platform [14] and found that SOCS1 was downregulated in patients with cirrhosis and HCC and more significantly decreased in those with HCC (Figure 1A). Subsequently, we detected the mRNA expression level of SOCS1 in 159 pairs of HCC and adjacent normal tissues collected in our hospital and found that the expression pattern was consistent with that observed in tissues from the database (Figure 1B). Moreover, 71% of patients had different degrees of downregulation of SOCS1 expression in HCC (Figure 1C). In the TMA, we also observed consistent experimental results by using patient samples purchased from other research institutions (Figure 1D). Therefore, we examined the mRNA and protein expression of SOCS1 in QSG-7701 cells and 7 different HCC cell lines (SMMC-7721, MHCC-97H, Hep3B, HepG2, Huh7, HCC-LM3 and SK-Hep-1). As consistently shown by qRT-PCR and Western blot, SOCS1 was minimally expressed in most HCC cells (Figure 1E), indicating that the SOCS1 level is associated with the cancer suppressor phenotype in HCC cell lines. According to a previous study, methylation of SOCS1 is an important factor in the decrease of its expression. We examined the degree of methylation of SOCS1 in QSG-7701 cells and 7 HCC cell lines. Compared to QSG-7701 cells, all the HCC cell lines except SMMC-7721 were found to have a measurable degree of methylation (Figure 1F). After that, we selected three HCC cell lines and used lentivirus to stably overexpress SOCS1 in these lines, and the overexpression efficiency was verified prior to execution of the subsequent experiments (Figure 1G).

### Overexpression of SOCS1 inhibited cell proliferation in vitro and in vivo

**Figure 2:**
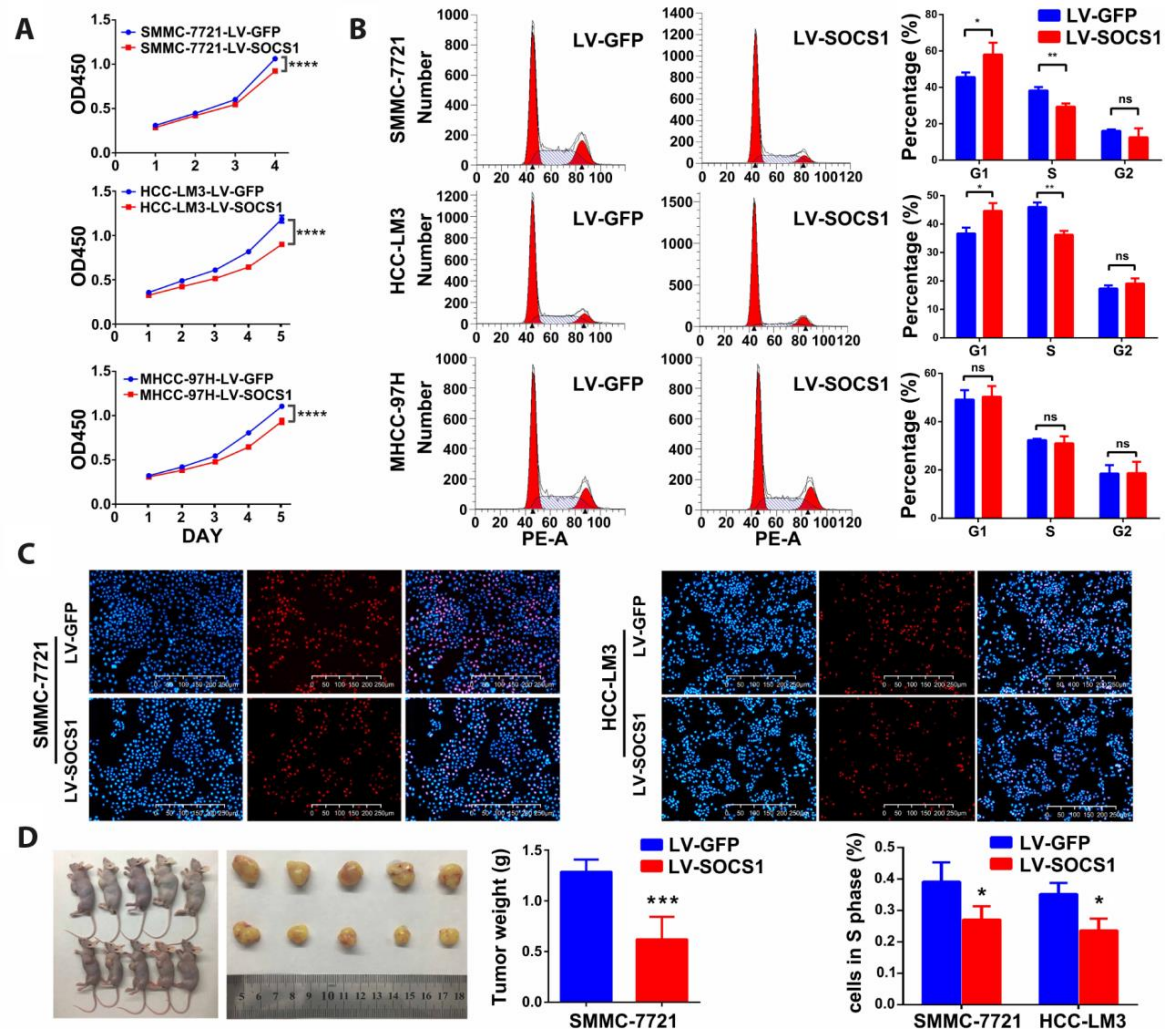
**SOCS1 inhibited HCC cell proliferation in vitro and in vivo.** (**A**) Cell viability was analysed by the CCK-8 assay. ****P <0.0001. (**B**) Representative FACS images of SMMC-7721, HCC-LM3 and MHCC-97H cells infected with LV-GFP or LV-SOCS1. * P <0.05, ** P <0.005. (**C**) Representative images of EdU incorporation assays in SMMC-7721 and HCC-LM3 cells. * P <0.05. (**D**) LV-SOCS1-transfected SMMC-7721 cells exhibited significantly reduced tumour volume and weight compared to those in control cells. ***P <0.001.

As shown in Figure 2A, the CCK-8 assays indicated that overexpression of SOCS1 markedly decreased the viability of all three HCC cell lines. To analyse the growth inhibition mechanism of SOCS1 in HCC, we used flow cytometry to detect the proportion of cells in each phase. We found that SOCS1 overexpression in SMMC-7721 and HCC-LM3 cell lines resulted in a significant increase in the proportion of cells in G1 phase and a significant decrease in the proportion of cells in S phase, indicating that there may be a block at G1-S transition. However, the MHCC-97H cell line did not exhibit G1 arrest in response to SOCS1 overexpression in our study (Figure 2B). Through the EdU experiment, we confirmed that SOCS1 was related to inhibition of the cell cycle progression of SMMC-7721 and HCC-LM3 cells (Figure 2C). To determine whether SOCS1 can also inhibit tumours in vivo, we investigated the effect of SOCS1 on tumour growth in immunodeficient nude mice. SOCS1-transfected SMMC-7721 cells or negative control cells were subcutaneously injected into mice (five animals per group). After six weeks, the mice were sacrificed, and their tumours dissected. Compared with the control tumours, tumours derived from cells with SOCS1 overexpression presented an apparent decrease in tumour size and weight (Figure 2D).

### Overexpression of SOCS1 blocked G1-S transition in SMMC-7721 and HCC-LM3 cells by repressing Rb phosphorylation

**Figure 3:**
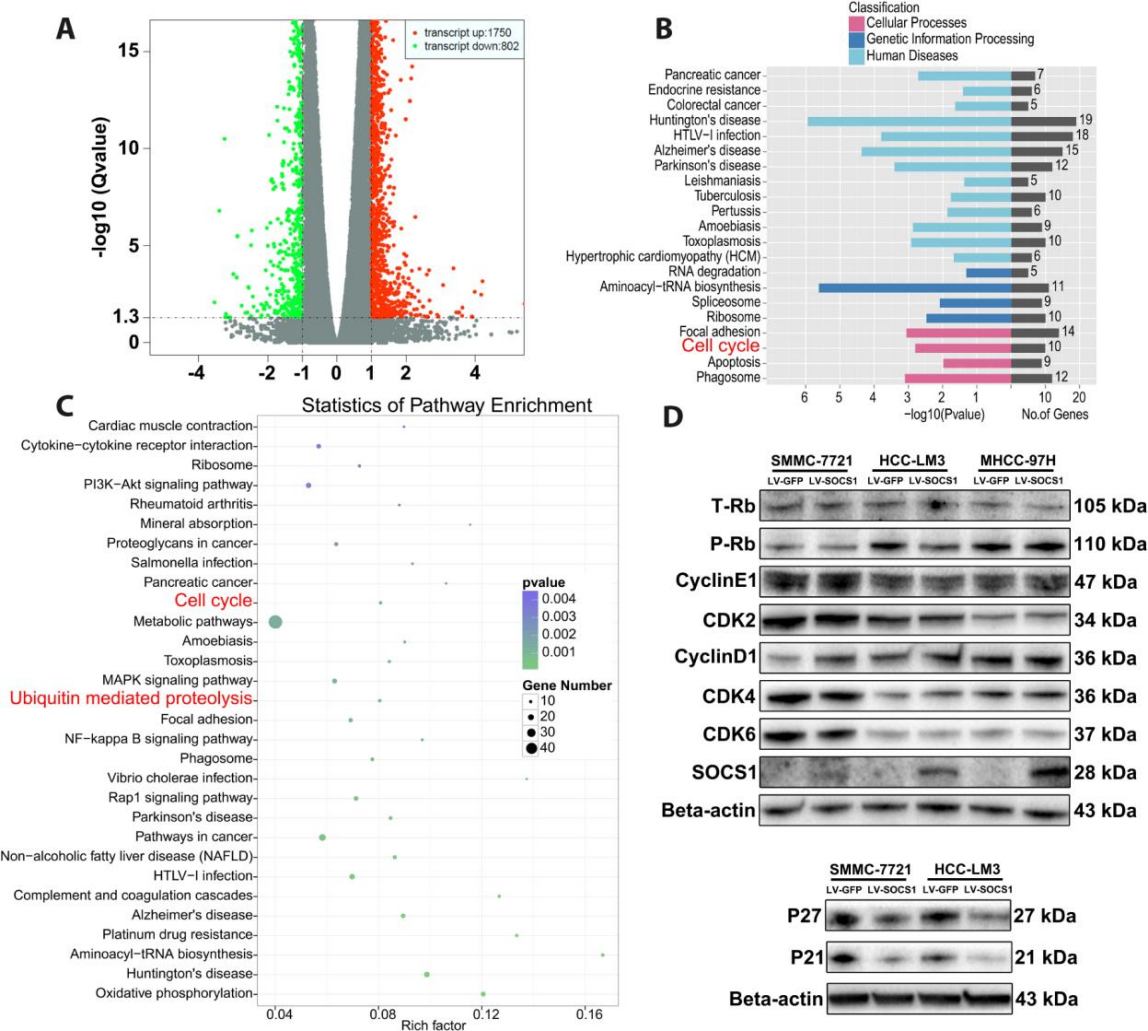
**The effects of SOCS1 overexpression on cell cycle progression.** (**A**) Transcriptome sequencing analysis showed 2552 differentially expressed genes (**B**, **C**) KEGG analysis indicated that a variety of cancers and processes relating to cell cycle and ubiquitin regulation were affected by SOCS1. (**D**) Western blot analysis revealed the effect of SOCS1 on the expression of cyclin proteins and cyclin-dependent kinases.

Transcriptome sequencing was used to determine the mechanism underlying SOCS1-medaited inhibition of HCC proliferation. Compared with control cells, HCC-LM3 cells transfected with a SOCS1 overexpression vector had a total of 2552 differentially expressed genes, including 1750 upregulated genes and 802 downregulated genes (Figure 3A). Furthermore, GO ontology analysis indicated that differential SOCS1 expression was associated with pancreatic cancer and colorectal cancer and affected cellular processes related to the cell cycle, apoptosis and phagosomes (Figure 3B). Pathway enrichment analysis not only suggested that SOCS1 was closely related to cancer but also showed that SOCS1 had a regulatory effect on the cell cycle. At the same time, ubiquitin, a key factor affecting the expression of cyclins, came into our view (Figure 3C). We verified by Western blot that SOCS1 overexpression reduced the levels of phosphorylated Rb in SMMC-7721 and HCC-LM3 cells. However, there was no significant change in the levels of CyclinE1, CDK2, CDK4 or CDK6. Consistent with the previous flow cytometry results, there was no significant change in the cell cycle-related proteins in MHCC-97H cells. To our surprise, CyclinD1 expression was upregulated after overexpression of SOCS1. Therefore, we measured the expression of the genes P21 and P27, which regulate the of CyclinD1 expression. It was surprising that downregulation of P21 and P27 expression was observed (Figure 3D), which was inconsistent with our original assumption.

### P21 was degraded by ubiquitin

**Figure 4:**
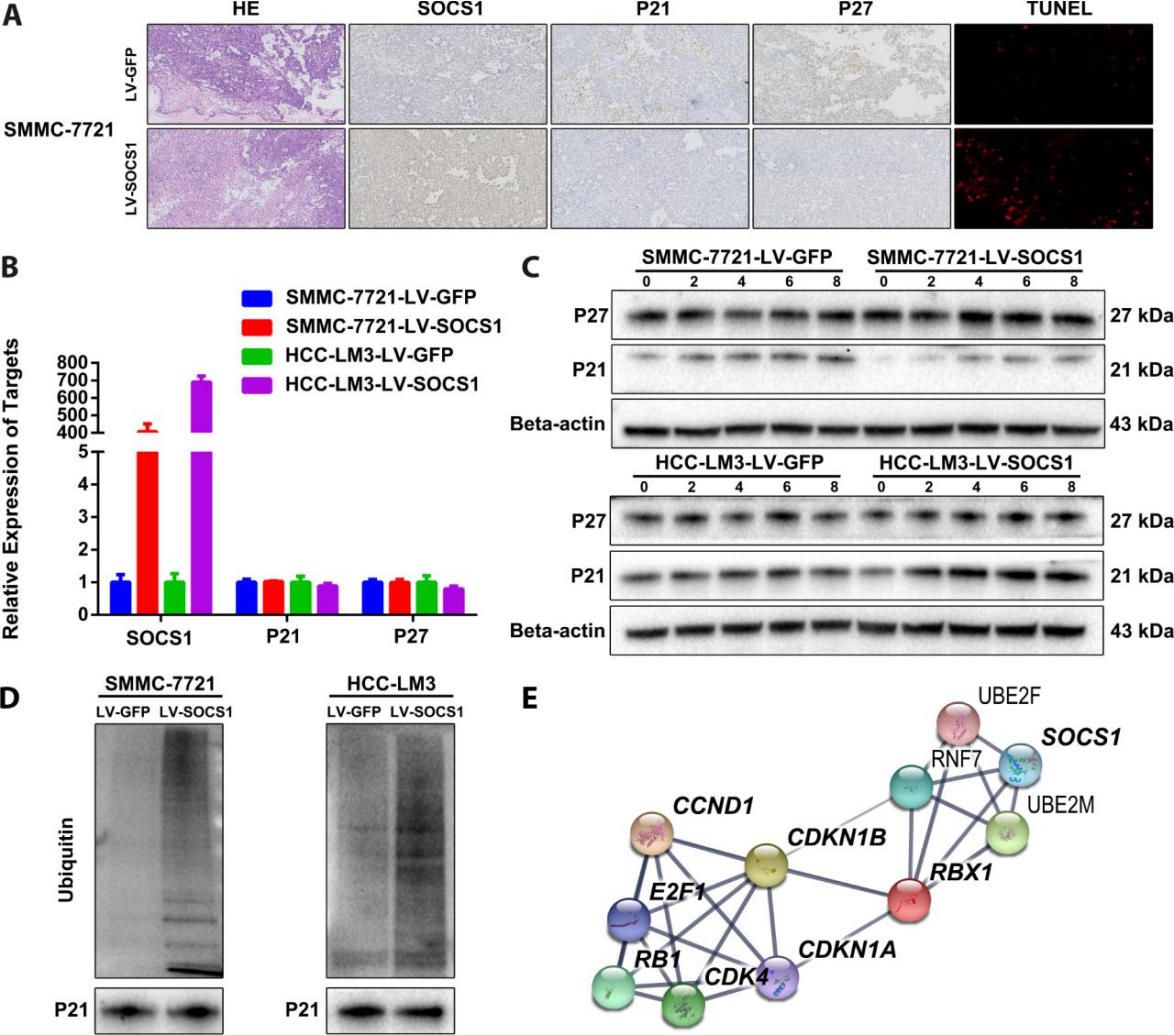
**SOCS1 overexpression degraded P21 via ubiquitin.** (**A**) Histochemical staining and TUNEL detection of tumour-bearing tissues in nude mice. (**B**) The mRNA levels of P21 and P27 are not regulated by SOCS1. (**C**) The ubiquitin inhibitory effect of 20 μM MG-132 (a proteasome inhibitor) on SMMC-7721 and HCC-LM3 cells was observed at 2 hours, 4 hours, 6 hours and 8 hours. (**D**) Detection of the difference in ubiquitination of P21 after overexpression of SOCS1 by Co-IP. (**E**) Interaction network between SOCS1, ubiquitin proteins, cyclin proteins and cyclin-dependent kinases.

We confirmed the changes in P21 and P27 expression in tumour sections collected from nude mice. As expected, tumours derived from SOCS1-transfected SMMC-7721 cells presented decreased p21 and p27 expression compared tumours derived from control cells (Figure 4A). However, we did not observe changes in P21 and P27 in the transcriptome sequencing results, which was confirmed by qRT-PCR (Figure 4B). When combining these results with those of the pathway enrichment analysis and the structural particularity of SOCS1, we speculated that P21 and P27 may be degraded by ubiquitin. We used 20 μM MG-132 (a proteasome inhibitor) in a time course and found that MG-132 reduced the protein degradation of P21 (there is no significant difference in P27), and the effect was most pronounced at 6 hours (Figure 4C). Therefore, we collected protein from SMMC-7721 and HCC-LM3 cells 6 hours after MG-132 treatment for Co-IP detection of ubiquitin and confirmed that the ubiquitination of P21 increased in cells with SOCS1 overexpression (Figure 4D). We input the ubiquitin proteins that were indicated by transcriptome sequencing and the proteins with differential expression into the STRING analysis tool [15] and obtained a protein-protein interaction network (Figure 4E). This assessment further confirmed that SOCS1 regulates the cell cycle by recruiting ubiquitin to degrade P21.

### Degradation of P21 led to a decrease in the stability of the CyclinD1/CD4 complex in the nucleus

**Figure 5:**
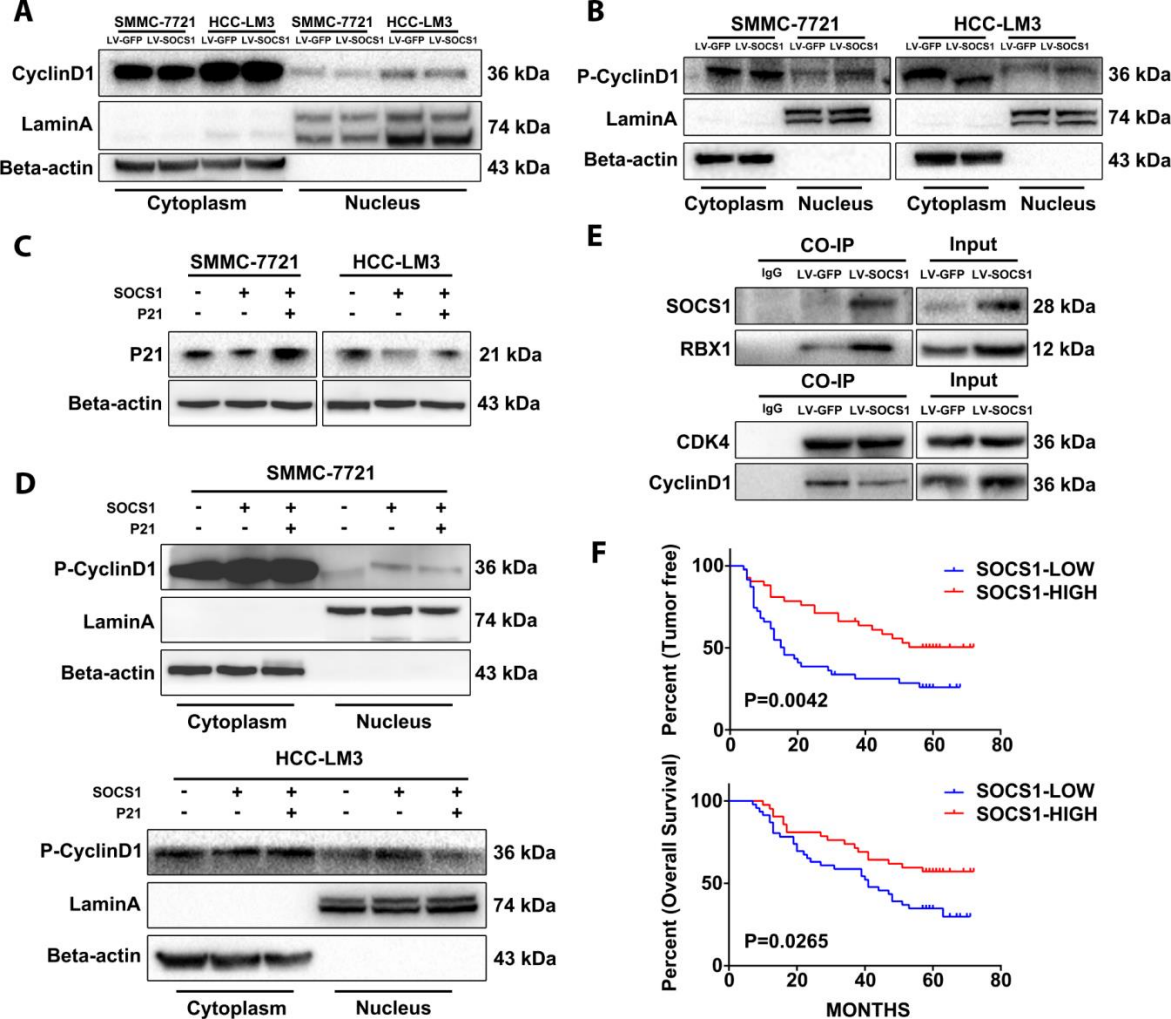
**The stability of CyclinD1 and CDK4 in the nucleus limits the cell cycle progression of the tumour.** (**A**) The amount of total CyclinD1 in the nucleus is not upregulated. (**B**) Effective reduction in CyclinD1 levels in the nucleus. (**C**, **D**) P21 can reduce the levels of phosphorylated CyclinD1 in the nucleus and stabilize its CyclinD1 binding to CDK4. (**E**) Co-IP confirmed that SOCS1 recruited ubiquitin and reduced the stability of CyclinD1/CDK4 complexes. (**F**) The high level of SOCS1 expression indicates low recurrence and better prognosis in patients with HCC.

The literature states that P21 has dual functions, and it plays an important role in promoting the nuclear stability of the CyclinD1/CDK4 complex. The interaction between CyclinD1 and CDK4 in the nucleus is the key factor in the phosphorylation of Rb and the progression of cycle to S phase, and phosphorylation of CyclinD1 halts this progression. Therefore, we extracted the cytoplasmic and nuclear protein fractions from SMMC-7721 and HCC-LM3 cells to analyse whether the increase in total CyclinD1 protein has an effect on the cell cycle. We found that the nuclear CyclinD1 in HCC-LM3 cells decreased after SOCS1 overexpression, and the level of phosphorylated CyclinD1 was higher than that in the control group (Figure 5A, 5B). The level of phosphorylated CyclinD1 in the nucleus was also increased in SMMC-7721 cells (Figure 5B). Different from the SOCS1 transfection procedure, we transfected plasmid expressing P21 into the two cell lines and detected the level of phosphorylated CyclinD1 in the nucleus (Figure 5C). It was found that the increased phosphorylation of CyclinD1 after overexpression of SOCS1 was partially reversed by overexpression of P21 (Figure 5D). Finally, we confirmed by Co-IP the binding of SOCS1 to the ubiquitin-related protein RBX1 (Figure 5E) and used CDK4 as a reference standard to detect whether there was a difference in the binding of CyclinD1 when the difference in CDK4 expression was very small. As expected, there was a significant decrease in the level of CDK4-bound CyclinD1 in the group of cells that overexpressed SOCS1 (Figure 5E).

### Association between SOCS1 expression and clinicopathological parameters of human HCC

To investigate whether SOCS1 is associated with clinical parameters in patients with HCC, we measured SOCS1 expression in an independent group of 90 HCC patients and conducted an extensive clinical follow-up. Clinicopathological correlation analysis showed that there was no significant correlation between the high SOCS1 expression group and the low SOCS1 expression group with regard to age, sex, preoperative AFP level, histopathological grade and tumour number; in fact, the only correlated observed was with tumour size (Table 1). Next, we explored whether SOCS1 could be an important molecular marker in predicting the clinical outcome of HCC patients and found that the tumour-free survival in HCC patients with high SOCS1 expression was significantly higher than that in patients with low SOCS1 expression (P = .0042, Figure 5F). The overall survival of the SOCS1 high expression group was also better than that of the SOCS1 low expression group (P = .0265, Figure 5F). In summary, when combined with clinical data, SOCS1 expression can be used as an indicator to predict the recurrence of HCC, which in turn may affect the overall survival of patients.

**Table 1:** Clinicopathological correlation of SOCS1 expression in human HCC.

**Variables**	**Tumor SOCS1 expression**		**P value^a^**
	Low	High	
Age			
<50 years	22	20	0.833
≥50 years	23	25	
Gender			
Female	7	9	0.784
Male	38	36	
Preoperative AFP level			
<400 ng/mL	27	30	0.662
≥400 ng/mL	18	15	
Histopathologic grading			
Well+moderately	28	30	0.826
Poorly	17	15	
Tumor size			
<5 cm	18	30	0.020
≥5 cm	27	15	
Tumor number			
Single	37	37	1.000
Multiple	8	8	

AFP: alpha-fetoprotein

HCC patients receiving surgery were segregated into SOCS1-high/low expression groups (calculation of cut-off by Immunohistochemistry).

^a^ Statistical analyses were performed with chi-square test.

## DISCUSSION

The SOCS family consists of eight intracellular proteins: SOCS 1-7 and CIS [16]. All these proteins possess an SH2 domain, a C-terminal SOCS box, an N-terminal extended SH2 subdomain and a variable N-terminal region. The SOCS box can recruit factors to form the E3 ligase complex, which marks the target protein for ubiquitination and consequently results in proteasome degradation [17, 18]. SOCS1 methylation may be used as a tumour suppressive factor in HCC [19–21], as similar reports observed this phenomenon in a variety of other tumours [22–28]. Based on the downregulation of SOCS1 expression in liver cancer specimens from the Oncomine database and the detection of SOCS1 in primary clinical specimens, we propose that SOCS1 plays an important role in the occurrence and development of HCC.

We first demonstrated that SOCS1 was downregulated in HCC tissues and that this downregulation may be related to increased levels of methylation. The CCK-8 assay showed that SOCS1 inhibited the overall activity of HCC cells. This may be due to either inhibition of cell proliferation or increased apoptosis, resulting in a decline in cell activity. To further explain this phenomenon, we carried out flow cytometry to measure the cell cycle distribution and found that the G1-S transition was inhibited in two kinds of HCC cell lines. We further confirmed by EdU staining of two cell lines that SOCS1 could partially block the cell cycle progression of HCC cells in G1 phase. To verify whether SOCS1 can also inhibit the proliferation of cancer cells in vivo, we injected SMMC-7721 stably overexpressing SOCS1 in immunodeficient nude mice, and the tumour volume and tumour growth rate of the SOC1 overexpression group were smaller than those of the control group. Next, we analysed transcriptional group sequencing in HCC-LM3 cells and found that the expression of genes related to the cell cycle and ubiquitin pathway was significantly changed. Because dysfunction of the cell cycle is common in tumours [5], we verified the key proteins in G1 phase by Western blotting, adding several related proteins that were not indicated in the sequencing results. It was found that the phosphorylation of Rb was inhibited by SOCS1, which led to reduced exposure of the E2F1 transcription factor and an inability to activate transcription, resulting in cell cycle arrest at the G1-S transition [29]. However, we accidentally found that CyclinD1 expression increased and that P21 and P27 expression decreased after SOCS1 overexpression. Based on the tumours resected from nude mice in vivo, we confirmed that P21 and P27 expression was downregulated. In this process, we also found a large area of necrosis in HE staining and confirmed with the TUNEL assay that SOCS1 can partially promote the apoptosis of HCC cells.

Upon consulting the literature, we realized that P21 and P27 have functions in promoting cancer: the first being their role in stabilizing CyclinD1 activity and localization, and the second suggesting that P21 exerts an anti-apoptotic effect [30–34]. These two pieces of evidence are in line with the results we have obtained. However, we only focused on the regulation of the cell cycle, especially relating to the stability of the CyclinD1/CDK4 complex in the nucleus [35]. While detecting the mechanism by which P21 and P27 are downregulated, we analysed the active components of CyclinD1 and the degree of CyclinD1 binding to CDK4. The transcriptome analysis indicated no changes in P21 and P27, and PCR did not detect an effect of P21 and P27 on CDK4 mRNA levels. Combined with the pathway enrichment analysis and the special domain SOCS box on SOCS1, we speculated that the ubiquitin degradation pathway may be the mechanism controlling the downregulation of P21 and P27 proteins. By assessing the temporal effect of the proteasome inhibitor MG-132, we confirmed that the degradation of P21 decreased with the use of MG-132, and the effect was the optimal after 6 hours of exposure. The results of the Co-IP experiment also showed that the levels of ubiquitinated P21 increased significantly after SOCS1 overexpression. We input several key molecules identified in this study into a STRING protein interaction analysis and obtain a clear network of protein-protein interactions, which is consistent with our conjecture. At the same time, through the protein analysis of the cytoplasmic and nuclear fractions, we found that the amount of CyclinD1 in the nucleus did not increase but that the levels of the inactive form (i.e., phosphorylated CyclinD1) increased. To verify whether this effect occurs upstream of P21, we transfected the P21 plasmid into cells with low P21 cell lines to artificially increase the protein expression of P21. We observed that the inactivation of CyclinD1 in the nucleus is partially reversed upon ectopic P21 expression.

**Figure 6:**
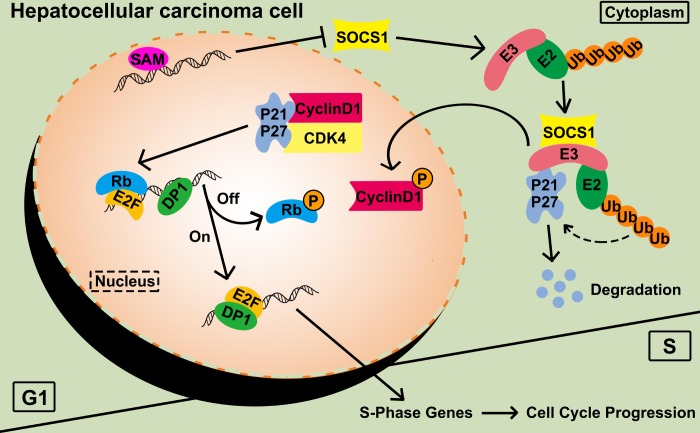
**Functional diagram representing the function of SOCS1 in HCC.** SAM: S-adenosyl methionine; P: phosphorylation; Ub: ubiquitination.

To connect the predicted pathways, we confirmed the binding of SOCS1 to the ubiquitin E3-related protein RBX1 by Co-IP. Equal amounts of CDK4 were used as a loading control to detect the amount of CDK4-bound CyclinD1 after SOCS1 overexpression. The interaction between CyclinD1 and CDK4 was weakened in the SOC1 overexpression group compared with the control group. The ubiquitin recruitment activity of SOCS1 leads to the degradation of P21, which mitigates its function (Figure 6).

We focused on the inhibition of proliferation of HCC and found that high levels of SOCS1 expression are related to a good prognosis of HCC patients and inhibit G1-S transition of HCC cells through the P21 -CyclinD1/CDK4-Rb pathway. It has been confirmed that P21 does not always function as tumour suppressor molecule in cancer. The study highlights the mechanism by which SOCS1 suppresses the growth of HCC, which is expected to be targeted and implemented as an anticancer drug in combination with other treatments.

## MATERIALS AND METHODS

### Clinical sample collection

In all, 159 pairs of HCC tissues and adjacent normal tissues were obtained from the First Affiliated Hospital of Zhejiang University. The tissue microarray (TMA) containing 90 pairs of tumours and matched adjacent tissues was purchased from Shanghai Outdo Biotech (Shanghai, China).

### Cell culture

One normal hepatocyte cell line (QSG-7701) and seven HCC cell lines (SMMC-7721, MHCC-97H, Hep3B, HepG2, Huh-7, HCC-LM3 and SK-Hep-1) were obtained from the Cell Bank of Chinese Academy of Sciences (Shanghai, China). All cells were cultured in RPMI 1640 medium (Biological Industries, Israel) or Minimum Essential Medium (Biological Industries, Israel) supplemented with 10% FBS (Biological Industries, Israel) at 37 °C in an incubator containing 5% CO2 (Thermo Scientific, USA).

### RNA isolation and quantitative RT-PCR

Total RNA was extracted from cell lines or HCC specimens using TRIzol reagent (Invitrogen). The PrimeScript RT-PCR kit (TaKaRa Biotechnology, Dalian, China) was used for reverse transcription. A SYBR Green PCR kit (Roche) was used for quantitative RT-PCR with a Bio-Rad PCR instrument (CA, USA). The primers used are listed as follows: GAPDH forward: GGAGCGAGATCCCTCCAAAAT; GAPDH reverse: GGCTGTTGTCATACTTCTCATGG; SOCS1 forward: CCTGCGGATTCTACTGGGG; SOCS1 reverse: ACGCTAAGGGCGAAAAAGCA; CDKN1A (P21) forward: GCAGACCAGCATGACAGATTT; CDKN1A (P21) reverse: GATGTAGAGCGGGCCTTTGA; CDKN1B (P27) forward: ACCTGCAACCGACGATTCTTCTAC; CDKN1B (P27) reverse: GCTTCATCAAGCAGTGATGTATCTG.

### Western blotting

Samples were lysed using RIPA buffer (Thermo Scientific, USA). A BCA Protein Assay Kit (Thermo Scientific, USA) was used to detect the protein concentrations. A total of 30 μg of protein was loaded on 10% SDS-PAGE (Life Technology, USA) for separation and then transferred to 0.45 μm or 0.2 μm PVDF membranes (Millipore, USA). After the membranes were blocked with 5% skim milk for 2 hours, they were cut, and the strips were incubated with appropriate primary antibody (1:2000) at 4 °C overnight. Subsequently, the membranes were washed and incubated with the corresponding HRP-conjugated secondary antibody (1:4000). Protein expression was detected by EZ-ECL (Biological Industries, Israel). The antibodies used in this paper are listed below.

Primary antibodies targeting beta-actin, LaminA, SOCS1, CDK4, CyclinD1, Total-Rb, CyclinE1, CDK2, CDK6, p21, p27, IgG, RBX1, and ubiquitin were purchased from Abcam, UK. Primary antibodies targeting phosphorylated Rb and phosphorylated CyclinD1 were purchased from Cell Signaling Technology, USA.

### DNA extraction and methylation detection

A Blood and Cell Culture DNA Midi Kit was purchased from QIAGEN, Germany, and a NanoDrop 2000 was used to detect the quality of DNA. An EZ DNA Methylation-Gold Kit (ZYMO, CA, USA) was used to transform the unmethylated cytosine (C) residues in genomic DNA into thymine (T) residues. The DNA methylation level at the CpG site of SOCS1 was determined by methyl target sequencing (Genesky Biotechnology Inc., Shanghai, China), which is a multi-target CpG methylation analysis method based on next-generation sequencing.

### Lentivirus and plasmid infection

The LV-SOCS1 lentivirus and control lentivirus encoding LV-GFP were constructed by GeneChem (Shanghai, China). HCC cell lines, including MHCC-7721, MHCC-97H and HCCLM3, were infected with lentivirus at a multiplicity of infection (MOI) of 30 pfu per cell. After infection, 10 μg/ml puromycin (Sigma-Aldrich, USA) was used to select stably transduced cells.

Plasmid containing SOCS1 and empty vector were purchased from GeneChem (Shanghai, China). Lipofectamine 3000 (Invitrogen) was used to transfect cells.

### Cell viability and EdU assay

Cell viability was detected with a Cell Counting Kit-8 Assay (Dojindo, Japan). Transfected HCC cells were seeded into 96-well plates (2 × 10^3^/ well) in 200 μl of medium. Supernatants were removed after the indicated number of days, after which 20 μl of CCK-8 and 180 μl of medium were mixed and added to each well. The plates were incubated for 1 hour, and then we measured the absorbance at 450 nm of each well with a microplate reader (BioTek, USA).

To determine the rates of DNA synthesis, transfected HCC cells in the logarithmic growth phase were seeded into 96-well plates (5× 10^2^/ well) in 200 μl of medium and subjected to an EdU Apollo 567 imaging kit (RiboBio, China).

### Cell cycle analysis

Transfected HCC cells were digested and fixed in 75% pre-cooled ethanol at 4 °C for 1 day. After the cells washed, they were resuspended with the Cell Cycle Staining Kit (Multi Sciences, China) at room temperature. The cell cycle distribution was determined using flow cytometry (FACS LSRII, BD Bioscience, USA).

### Nuclear and cytoplasmic protein extraction

The Nuclear and Cytoplasmic Extraction Reagents Kit (Thermo Scientific, USA) was used according to the manufacturer’s instructions to extract and separate the nuclear and cytoplasmic proteins from transfected SMMC-7721 and HCC-LM3 cells.

### Tumour xenograft model

Four-week-old immunodeficient nude male mice were divided randomly into two groups. Approximately 4×10^6^ transfected SMMC-7721 or HCC-LM3 cells were subcutaneously injected into the mice (in each case, 4×10^6^ cells suspended in 100 μL PBS). After six weeks, all mice were sacrificed to measure the tumour weight and volume before the tumours were harvested for immunohistochemical analysis.

### Co-immunoprecipitation and ubiquitination assays

Co-IP assays were performed as previously reported [36]. Lysates from transfected HCC-LM3 were used for immunoprecipitation with SOCS1, CDK4 and IgG primary antibodies on protein A/G mix beads (Thermo Scientific, USA) overnight. Next, 20 μΜ protease inhibitor (MG-132, Sigma-Aldrich, USA) was added to the medium of transfected SMMC-7721 and HCC-LM3 cells 6 hours before lysis. Cell lysates were used for immunoprecipitation with p21 primary antibody on protein A/G mix beads overnight to detect p21 ubiquitination.

### Immunohistochemistry

Immunohistochemical analysis was carried out as we reported earlier [37]. Tumours harvested from immunodeficient nude mice were immunostained with anti-SOCS1 (1:50), anti-p21 (1:100) and anti-p21 (1:100) antibodies, while samples from the tissue microarray were immunostained with anti-SOCS1 (1:50) antibody.

### Microarray analysis

Three biological replicates of HCC-LM3-LV-SOCS1 and HCC-LM3-LV-GFP cells were used to detect differences in gene expression. The samples were tested by RiBo (RiboBio Co., Guangzhou, China), and a cDNA library was constructed and sequenced on an Illumina HiSeq 2500. The relevant Illumina analysis software was used to collect the original readings. The Kyoto Encyclopedia of Genes and Genomes [38] pathway annotation and Fisher’s Exact Test were analysed in the same way as we reported earlier [39].

### Statistical analysis

The independent t-test was used to analyse continuous data between the 2 groups. The chi-square test was used to analyse categorical data. The Kaplan–Meier method was used to analyse recurrence-free survival and overall survival, and the differences in survival between the groups were estimated by the log-rank test. All statistical analyses were performed using SPSS for Windows v.22.0 (SPSS, Chicago, IL) and GraphPad Prism 6.0 (GraphPad Software, La Jolla, CA). P < 0.05 was considered statistically significant.

### Ethical approval

The study protocol was approved by the ethics committee of the First Affiliated Hospital of Zhejiang University. All experiments containing animals according to protocols approved by Animal Care and Use Committee of Zhejiang University.
